# Linking TLR-7 Signaling to Downregulation of Placental P-Glycoprotein: Implications for Fetal Drug Exposure

**DOI:** 10.3390/pharmaceutics17060741

**Published:** 2025-06-05

**Authors:** Mario Riera-Romo, Eliza R McColl, Micheline Piquette-Miller

**Affiliations:** 1 Leslie Dan Faculty of Pharmacy, University of Toronto, Toronto, ON M5S 3M2, Canada; mario.rieraromo@mail.utoronto.ca; 2 Section of Infectious Diseases, Baylor College of Medicine, Houston, TX 77030, USA; eliza.mccoll@bcm.edu

**Keywords:** viral infections, autoimmune diseases, TLR-7, inflammation, transporters, P-glycoprotein

## Abstract

**Background/Objectives:** Activation of the Toll-like receptor 7 (TLR-7) plays an important role in the pathogenesis of many autoimmune diseases and viral infections. Although we have previously observed inflammation-mediated dysregulation of placental transporters, the role of TLR-7 has not been examined. Using the TLR-7 agonist, imiquimod (IMQ), we evaluated transporter expression in IMQ-treated pregnant rats and ex vivo in cultured rat placental explants. **Methods:** We administered 5 mg/kg (IP) of IMQ to pregnant Sprague Dawley rats on gestational day (GD) 14. The expression levels of inflammatory biomarkers and transporters were measured in maternal and fetal tissues by qRT-PCR and immunodetection methods, and effects on the placental proteome were assessed using LC/MS/MS. The involvement of TLR-7 was confirmed in rat placental explants. **Results:** IMQ administration resulted in *Irf7* induction and increased levels of IL-6, Tnf-α, and type-I/II interferon pathways in maternal liver and placenta, which is consistent with TLR-7 activation. Proteomic profiling revealed IMQ-mediated activation of pathways involved in immune response, vesicle trafficking, and oxidative stress. Significantly decreased placental, hepatic, and renal protein expression of P-glycoprotein (PGP) was seen in the IMQ group. Likewise, TLR-7 activation using single-stranded RNA resulted in an induction of inflammatory biomarkers and downregulation of PGP in rat placental explants. **Conclusions:** We demonstrated that the activation of TLR-7 signaling during pregnancy reduces the expression of PGP in placenta and maternal tissues. Further studies are warranted, as decreased protein expression could result in decreased activity and altered fetal exposure to its substrates.

## 1. Introduction

Physiological changes during pregnancy significantly influence drug disposition. However, ethical concerns often lead to the exclusion of pregnant individuals from clinical studies, limiting our understanding of pharmacokinetic alterations during this period [1,2]. Additionally, inflammatory diseases can affect drug absorption, distribution, metabolism, and excretion by altering the expression and activity of drug transporters and metabolizing enzymes [3,4]. Placental ATP-binding Cassette (ABC) efflux transporters play a crucial role in protecting the fetal compartment from xenobiotic accumulation [5]. Among these, breast cancer resistance protein (BCRP/ABCG2) and P-glycoprotein (PGP/ABCB1) are highly expressed on the apical membrane of placental syncytiotrophoblasts throughout gestation, limiting drug transfer into the placenta and fetal circulation [6,7,8]. These transporters also contribute to drug excretion in the intestine, liver, and kidney [9,10]. Additionally, multidrug resistance proteins (MRPs), including MRP1 (ABCC1) and MRP3 (ABCC3), may be involved in placental transport, though their precise roles remain less defined [11,12,13]. Previous research has shown that infection and inflammation during pregnancy modify the expression of drug transporters in the liver, kidney, and placenta, leading to significant changes in drug substrates [14,15,16].

Toll-like receptor (TLR)-7 signaling is an important part of the innate immune response against single-stranded RNA (ssRNA) viruses such as Influenza, Hepatitis C, HIV and SARS-CoV-2 [17,18]. This receptor recognizes viral ssRNA and promotes the production of proinflammatory cytokines such as IL-6, IL-1β, and TNF-α, which create an inflammatory environment involved in immune cell recruitment, differentiation, and activation. TLR-7 also induces nuclear translocation of the interferon response factor (IRF)-7, which induces expression of type-I interferons such as IFN-α that are actively involved in immunoregulation and antiviral defense [19,20]. However, during chronic viral infections, continued activation of this pathway and the overexpression of proinflammatory cytokines are responsible for enduring symptoms and systemic complications [17,18]. Similarly, autoimmune diseases such as Sjögren’s syndrome and Lupus, which are characterized by the production of antinucleic acid antibodies (ANAs), are associated with chronic activation of the TLR-7 pathway, leading to sustained inflammation and tissue damage [21,22,23].

Many autoimmune diseases such as lupus have a much higher incidence in woman and their onset occurs at child-bearing age, thus increasing the likelihood of symptomatic disease during pregnancy [24]. Hormonal and physiological changes also make pregnant woman more susceptible to viral infections and disease flares, which impose serious risks for both maternal and fetal health [25]. In such scenarios, precise therapeutic management with optimal doses are essential to ensure the safety of both mother and baby. However, the impact of disease on maternal and fetal drug disposition is poorly understood for the majority of drugs. It has been demonstrated that inflammation can dysregulate drug transporter and metabolizing enzyme expression, which can impact drug disposition [26], but this has been understudied in the context of pregnancy. The TLR-7 agonist, imiquimod (IMQ), has been frequently used to stimulate disease-mediated activation of this pathway in vivo in preclinical models of lupus and viral-mediated inflammation [27,28,29]. In both pregnant and non-pregnant rodent models, systemic or topical administration of IMQ produced strong immunological responses characterized by elevations in proinflammatory cytokines and type-I interferons [29,30,31]. In the present work, we assessed the impact of TLR-7-associated inflammation during pregnancy on the expression of ABC efflux transporters, which are crucial to maternal and fetal drug disposition. To evaluate this, we activated TLR-7 in vivo in pregnant rats by IMQ administration. We also confirmed the involvement of TLR-7 signaling in transporter dysregulation through ex vivo experiments in rat placental explants.

## 2. Materials and Methods

### 2.1. Animal Model

Timed pregnant Sprague Dawley rats were purchased from Charles River Laboratories (Senneville, QC, Canada). Rats were maintained on a 12 h light–dark cycle with access to water and standard chow. On GD14, rats were given an intraperitoneal (IP) injection of the selective TLR-7 agonist imiquimod (IMQ; MedChemExpress, Monmouth Junction, NJ, USA) at a dose of 5 mg/kg dissolved in sterile, endotoxin-free water or endotoxin-free water alone (controls). At 6, 24, and 48 h post-IMQ (n = 4/group), animals were sacrificed. Placenta, liver, and kidney tissue were collected and snap-frozen in liquid nitrogen prior to storage at −80 °C for later analysis. Maternal blood was collected in BD Vacutainer tubes and centrifuged for 1430× *g* for 15 min at 4 °C to collect serum, which was subsequently snap-frozen and stored at −80 °C. Dose selection, administration route, and time of exposure were based on a previous study which examined the effects of IMQ administration on placental expression of amino acid transporters in rats [30]. Compared to controls, we did not detect any significant impact of IMQ on the number of viable fetuses, placental weight, or fetal weight. All animal experiments were performed according to the regulations of the Office of Research Ethics at the University of Toronto (AUP 20011917; 2 July 2022) and conducted according to guidelines of the Canadian Council on Animal Care.

### 2.2. Sexing Rat Embryos

Fetal sex was determined through qPCR amplification of the male-sex-determining region Y (*Sry)* in total DNA extracted from fetal tails, as previously described [32]. Fetal tails were first digested with Proteinase K overnight at 60 °C, after which potassium acetate was added to precipitate nucleic acids at −80 °C for one hour. Following centrifugation and rinsing, DNA was resuspended in nuclease-free water and subjected to PCR amplification for *Sry* and *Actb* (internal control) and visualized by gel electrophoresis. Placental sex was established according to its corresponding fetal sex for all embryos.

### 2.3. Rat Placental Explants

Placentas were isolated from untreated pregnant rats on GD 19 and rapidly washed with ice-cold PBS before preparing explants. Each placenta was dissected into 5 equivalent portions of tissue (~30–50 mg/explant) from maternal side of placenta and cultured in 24well plates at 37 °C, 21% O_2_, and 5% CO_2_ in Dulbecco’s Modified Eagle Medium (DMEM) supplemented with 5% FBS and PenStrep antibiotic. After a 24 h acclimation period, explants were treated for either 6 or 24 h with FBS-free DMEM media containing either IMQ (5 µg/mL) or ssRNA40 (1 or 5 µg/mL) (InvivoGen, San Diego, CA, USA); maternal serum was isolated from the 6 h IMQ-treated rats (10% serum) or media alone (controls). Media was supplemented with PenStrep (without FBS). Explants from the same placenta were used for paired experimental design and analysis of control (untreated) vs each treatment. After 6 or 24 h treatment periods, the explants were collected and processed for qPCR analysis. Expression of TLR-7-associated target genes and transporters were examined in treated and untreated explant samples. Experimental and treatment conditions were based on previous studies using freshly cultured rat and human placental explants [33,34,35,36,37,38].

### 2.4. Placental Proteomics

Using previously described methods [39,40], placental samples from control and IMQ-treated rats (n = 8 per group) were examined for global changes in the placental proteome profile in response to TLR-7 activation in control and IMQ-treated rats (n = 8 per group). Proteins were extracted from 200 mg of homogenized placental samples. Extracted proteins were digested with trypsin (bottom-up proteomics), and around 1 ug of peptides from each sample was loaded in duplicates onto the ThermoFisher Q-Exactive Orbitrap HPLC-MS/MS system using a C18 reverse-phase column. The data were exported and analyzed on MaxQuant software (version 2.2) using label-free quantification (LFQ) method. Output files from MaxQuant were analyzed in Perseus software (version 1.6) to obtain the ranked list of regulated proteins. Pathway enrichment analysis was performed in G-profiler. Heat map of differentially regulated proteins was performed in Perseus 1.6.

### 2.5. Real-Time Quantitative Polymerase Chain Reaction (RT-qPCR)

RNA was extracted from rat placentas, liver, and kidney using TRIzol, and real-time quantitative PCR was performed as previously described [14]. Briefly, total RNA was extracted, quantified, treated with DNase1, reverse-transcribed, and then amplified and quantified using Power SYBR Green (Thermo Fisher Scientific, Waltham, MA, USA) on the Bio-Rad CFX384 Touch PCR detection system (Bio-Rad, Hercules, CA, USA). Relative mRNA expression was calculated using the ΔΔCt method and normalized to *Gapdh*. Primer sequences are listed in Table S1.

### 2.6. Western Blot

Crude membrane and whole cell protein fractions were isolated from tissues via centrifugation, as previously described [29]. Protein samples were separated by SDS-PAGE gel electrophoresis and transferred to polyvinylidene difluoride membranes. Protein expression of PGP was measured by immunoblotting placental, hepatic, and renal tissue samples obtained at 48 h post-IMQ using the primary antibody D-11 (1:50) (sc-55510, Santa Cruz Biotechnology Inc., Dallas, TX, USA) followed by washing and incubation with the goat antimouse secondary antibody (1:75,000) (AP308PMI, Fisher Scientific, Toronto, ON, Canada). Expression was normalized to β-actin (AC-15, 1:100,000) (A1978, Millipore Sigma, Oakville, ON, Canada). Band intensity was quantified using ImageJ software 1.53e.

### 2.7. Statistical Analysis

Statistical analyses were performed using GraphPad Prism software version 8.0. Differences between groups were analyzed by Student’s unpaired *t*-test (for in vivo data) and paired *t*-test, as well as one-way ANOVA with Dunnet post-test (for rat placental explants). Proteomic data were statistically analyzed in Perseus software version 1.6, which uses a combination of *t*-test and ANOVA, in addition to Z score and principal component analysis or PCA, to find differently expressed proteins withing the dataset. Data are expressed as the as the average ± SEM relative to controls. *p* values less than 0.05 were considered significant (* *p* < 0.05, ** *p* < 0.01, *** *p*< 0.001). Experiments were done in triplicate and repeated at least twice.

## 3. Results

### 3.1. In Vivo Administration of IMQ Activates TLR-7-Mediated Inflammatory Response

**Figure 1:**
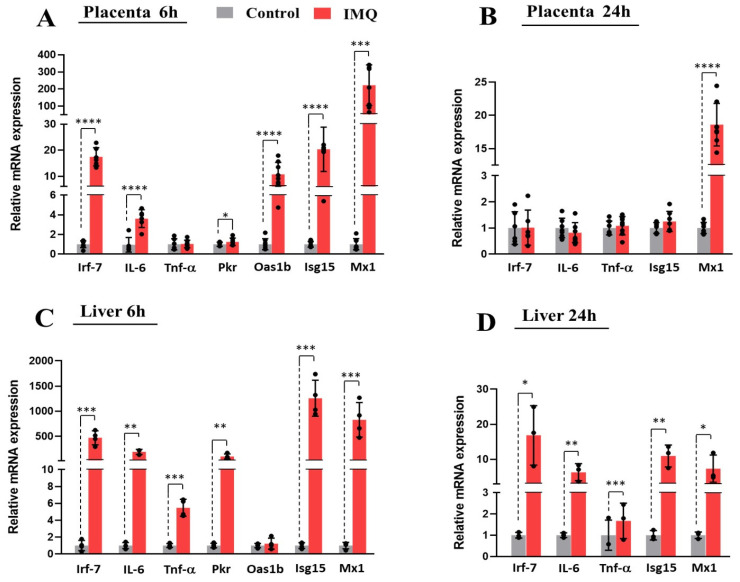
Effect of IMQ on transcript levels of TLR-7 biomarkers and IFN-α target gene expression in placenta (**A**,**B**) and liver (**C**,**D**). Animals were administered IMQ (5 mg/kg ip) on GD 14 and analyzed via qRT-PCR, as described in methods. Levels were normalized to *Gapdh* and are shown as mean ± SEM relative to controls. Statistical significance was determined using Student’s unpaired *t*-test (* *p* < 0.05, ** *p* < 0.01, *** *p* < 0.001, **** *p* < 0.0001).

Compared to the controls, the IMQ-treated dams demonstrated a significant 4.5-fold increase in serum protein levels of IFN-γ as well as pronounced increases in serum protein levels of several INF-γ-dependent chemokines [1], including interferon gamma-induced protein 10 (IP-10), Monocyte Chemoattractant Protein-1 (MCP-1), and Macrophage Inflammatory Protein-1 Alpha (MIP-1α) at 6 h after IMQ administration (Supplemental Figure S1). Trends of 1.5- to 3-fold increase in serum levels of IL-4, IL-5, IL-6, IL-17, and TNF-α were also seen, but these did not reach significance. The mRNA expression of various inflammatory markers was also evaluated in placentas and livers of pregnant rats after IMQ administration. A transient induction of the TLR-7 downstream gene *Irf7* was seen in the placentas and livers of IMQ-treated rats compared to the control rats, confirming TLR-7 activation (Figure 1). Likewise, the expression levels of *Il-6* and *Tnf-a* were increased in the livers of IMQ-treated dams at 6 h and 24 h post-injection, and *Il-6* expression was increased in the placentas of IMQ-treated dams at 6 h post-injection. As TLR-7 activation mediates increased production of IFN-α, we examined the placental and hepatic mRNA expression of four IFN-α target genes: RNA-dependent protein kinase (*Pkr*), 2′-5′ oligoadenylate synthetase 1B (*Oas1b*), interferon-stimulated gene 15 (*Isg15*), and Myxovirus resistance protein 1 (*Mx1*). The increased expression of *Pkr, Isg15*, and *Mx1* in both the placentas and livers of treated dams from 6 to 24 h, as well as higher placental levels of *Oas1b* at 6 h post-IMQ, indicate a strong induction of IFN-α activity in the IMQ group (Figure 1).

The transcript levels of these biomarkers were not significantly different between IMQ-treated dams and control dams at 48 h post-IMQ (Supplemental Figure S2). The impact of IMQ on *Irf* expression and other biomarkers of inflammation were compared between female and male placentas, and no sex-specific differences were found.

### 3.2. IMQ Alters the Placental Proteome and Activates Inflammatory Pathways

**Figure 2:**
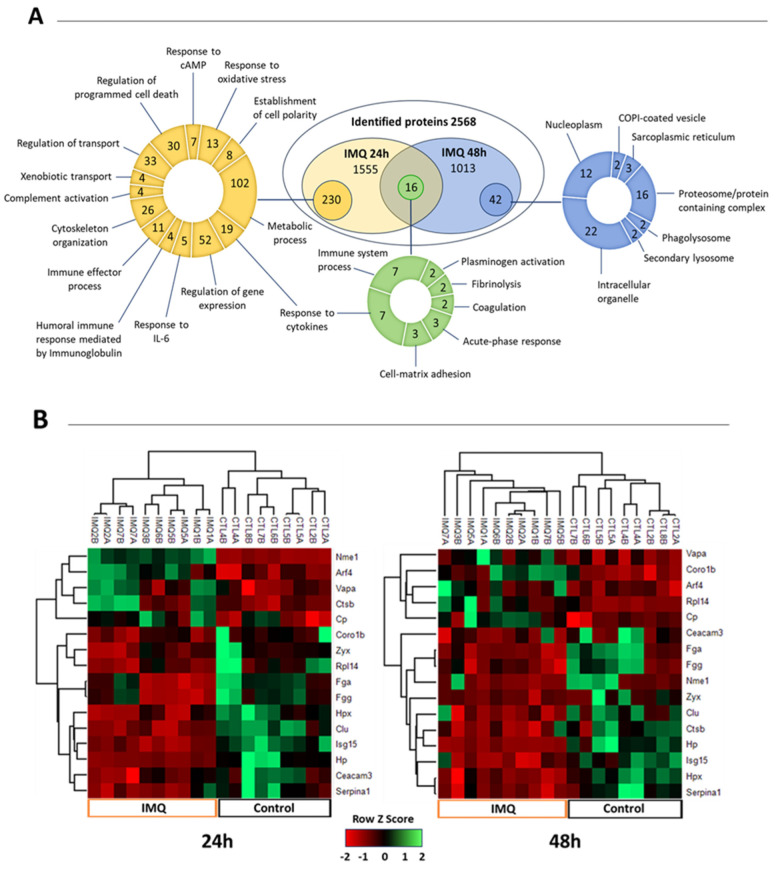

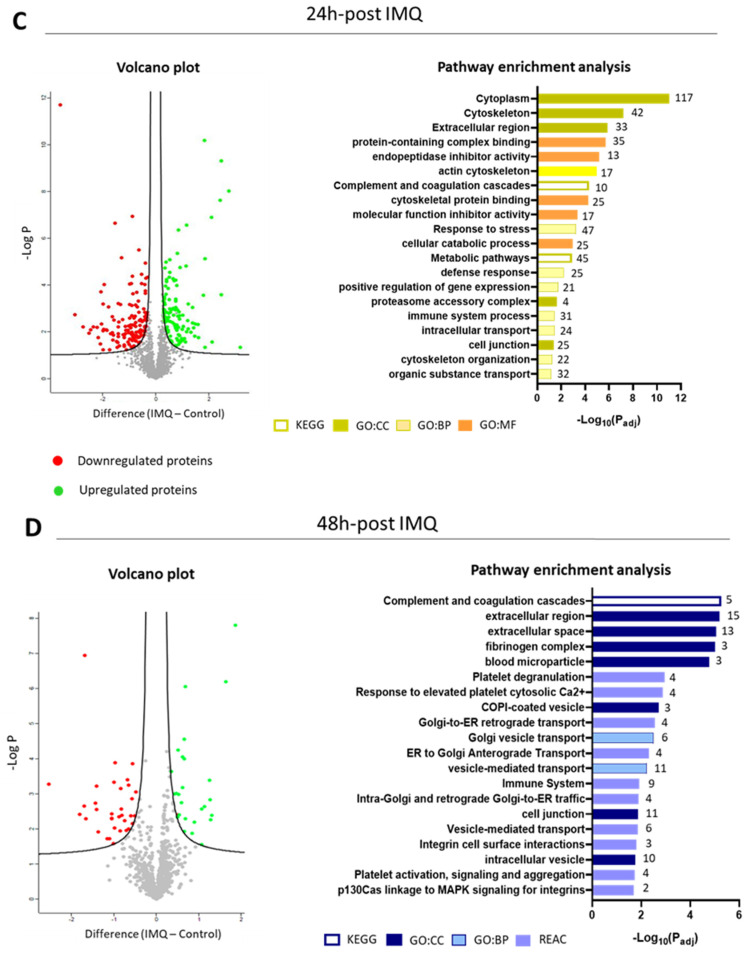
Impact of IMQ on placental proteome in pregnant rats. Placentas were collected from rats treated with 5 mg/kg of IMQ or saline (control) on GD 14, 24, and 48 h post-injection. Data were analyzed using MaxQuant 2.2 and Perseus 1.6. (**A**) Venn diagram representing the differences and the intersections of differentially modulated proteins from 24 h and 48 h datasets, including the main biological processes. (**B**) Heat map showing the differential expression between control and IMQ groups for the proteins that were modulated in both datasets based on Row Z Score and Hierarchical Analysis. Lower panels contain the Volcano Plot and the pathway enrichment analysis from G-profiler showing the top 20 regulated biological processes in response to IMQ exposure for 24 h (**C**) and 48 h (**D**). The numbers on top of each process indicate the number of genes identified in each biological function.

In order to expand the characterization of the inflammatory response and the activation of TLR-7-associated signaling pathways in the placenta, we performed a proteomic profiling of the placentas from control and IMQ-treated dams. In our study, 24 h and 48 h samples were chosen in order to best observe changes at the protein level. A total of 2568 proteins were identified between the datasets (1555 at 24 h and 1013 at 48 h). Significant findings include the differential regulation of 258 proteins at 24 h (121 upregulated and 134 downregulated) and 61 proteins at 48 h (27 upregulated and 34 downregulated) (Figure 2). A list of significant differentially expressed proteins from 24 and 48 h datasets with more than 10% change between the control and IMQ group is provided as Supplementary Material (Table S2).

**Figure 3:**
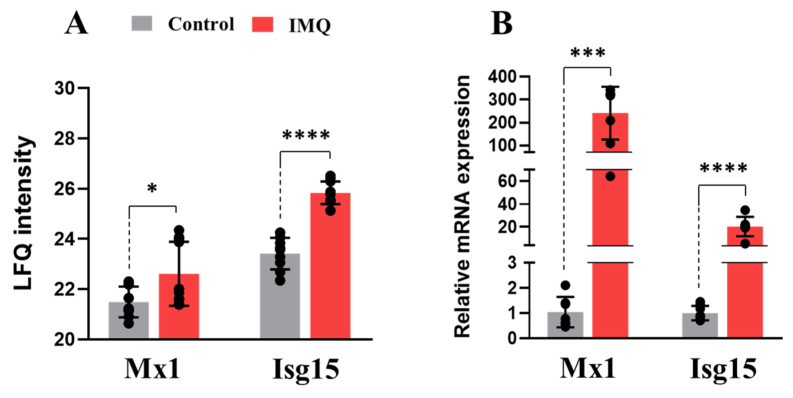
Relative abundance of INF-α downstream factors in IMQ-treated rat placentas. (**A**) Proteomic quantification of Mx1 and Isg15 by label-free quantification (LFQ) method in placentas from 48 h IMQ-treated rats. (**B**) Transcript levels of Mx1 and Isg15 on rat placentas 24 h post-IMQ. Rats were administered with 5 mg/kg of IMQ or saline (control) on GD 14. Whole-cell extraction of placental proteins and proteomic quantification by LC/MS/MS were performed as described in methods. RT-qPCR validation of proteomic targets was performed as previously described. Statistical significance was determined using Student’s unpaired *t*-test (* *p* < 0.05, *** *p* < 0.001, **** *p* < 0.0001).

Differentially expressed proteins in our datasets included clusterin, serpin1, various endopeptidases, and complement protein C3, indicating immune activation. The modulation of RNA-binding proteins and ribosomal proteins, as well as the downregulation of the eukaryotic translation initiation factor 4E (Eif4e), suggests the modulation of transcription and gene expression at 24 h post-IMQ. Some of these proteins were also found to be downregulated in the placenta of lupus patients in a previous proteomic study [41] (Table S2). Cytokine and IL-6 associated responses, regulation of gene expression, and programmed cell death were key modulated processes (Figure 2A,C). Likewise, the downregulation of antioxidant factors such as cytosolic non-specific dipeptidase or carnosine dipeptidase 2 (Cndp2), paraoxonase 1 (Pon1), and the copper-containing amine oxidase (Aoc1) indicates oxidative stress was another process associated with IMQ-induced inflammation. Proteins involved in vesicle trafficking and lysosomal processing were also differentially expressed at 48 h post-IMQ. Commonly induced proteins at both time points included acute phase proteins and immune factors such as fibrinogen A (Fga/Fgg), haptoglobin (Hp), and hemopexin (Hpx) (Figure 2B). The INF-α downstream targets Isg15 and Mx1, implicated in host response to infection and inflammation, were also upregulated, particularly after 24 h of IMQ exposure (Figure 2, Table S2). Both targets were validated by RT-qPCR (Figure 3). This correlates with the gene expression data and demonstrates the activation of INF-α-mediated pathways in the placentas of IMQ-treated dams.

Pathway enrichment analysis predicted the regulation of coagulation cascade, proteolytic activities, signal transduction, immune response activation, cell adhesion, metabolic processing, cytoskeleton rearrangement, and vesicle-mediated transport as the main biological processes impacted by IMQ treatment in the placentas (Figure 2C,D).

### 3.3. In Vivo Administration of IMQ Reduces Expression of PGP

**Figure 4:**
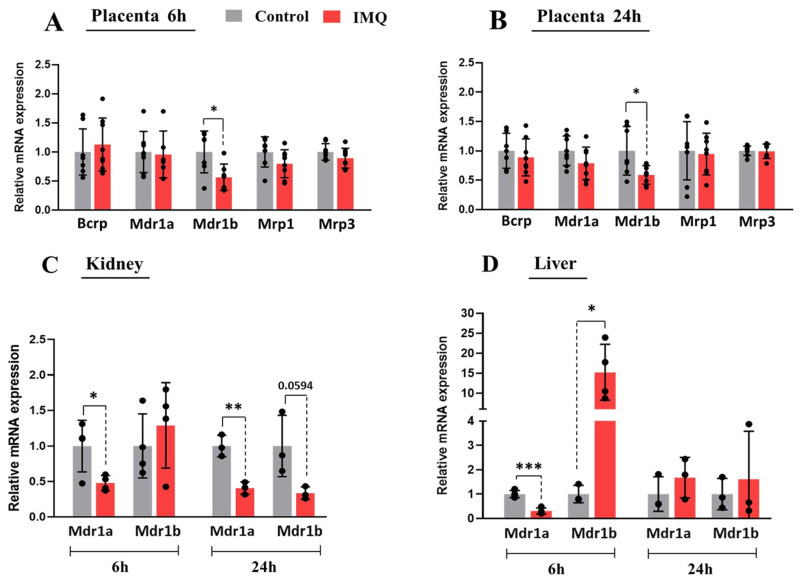
Impact of imiquimod (IMQ) on ABC transporter expression in pregnant rats. Transcript levels of transporters in placenta at 6 h (**A**) and 24 h (**B**) post-IMQ. Transcript levels of Mdr1a and Mdr1b in liver (**C**) and kidney (**D**) 6 and 24 h after IMQ injection. Expression of transporters was measured via qRT-PCR as described in methods. Statistical significance was determined using Student’s unpaired *t*-test (* *p* < 0.05, ** *p* < 0.01, *** *p* < 0.001).

**Figure 5:**
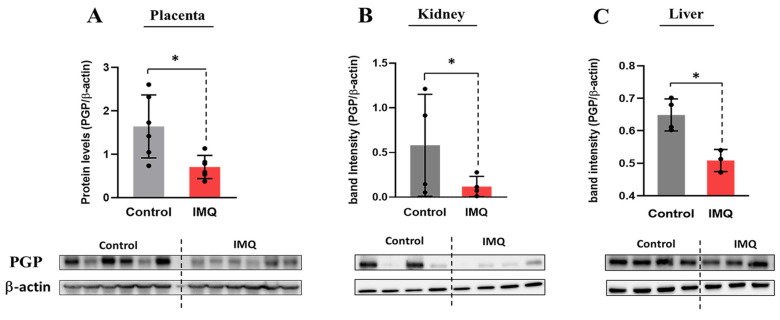
Impact of imiquimod (IMQ) on PGP protein levels in placenta (**A**), kidney (**B**), and liver (**C**) samples of pregnant rats 48 h post-injection. PGP was measured by immunodetection as described in methods and normalized to b-actin. Band intensity was measured using ImageJ software. Statistical significance was determined using Student’s unpaired *t*-test (* *p* < 0.05).

In rodents, PGP is encoded by the *Mdr1a* and *Mdr1b* transcripts. Compared to the controls, the mRNA expression of *Mdr1b,* but not *Mdr1a,* was significantly decreased by 50% at 6 and 24 h in placentas isolated from IMQ-treated dams (Figure 4A,B). Likewise, a significant 45% decrease in PGP protein expression was seen in placentas of the IMQ-treated group at 48 h (Figure 5A). IMQ did not significantly alter the transcript levels of *Bcrp, Mrp1*, or *Mrp3*. No sex-specific differences in the expression of *Mdr1a/b*, PGP protein, or IMQ-mediated changes were detected between female and male placentas.

As the expression levels of *Mdr1b* and PGP were decreased in the placentas of IMQ-treated dams, we further examined the mRNA and protein expression of PGP in their liver and kidney samples. IMQ treatment was associated with a significantly decreased protein expression of PGP in the kidney samples (Figure 5B) along with decreased mRNA levels of both *Mdr1a* and *Mdr1b* (Figure 4C). On the other hand, while the mRNA levels of *Mdr1a* were also decreased in the livers of IMQ-treated dams, the *Mdr1b* levels were increased (Figure 4D). These changes were associated with a significant reduction in the hepatic protein expression of PGP in treated dams (Figure 5C). No significant differences in the transcript levels of any transporters were seen at 48 h except for *Mdr1a*, which was reduced in the kidneys of the IMQ group (Supplemental Figure S3).

### 3.4. Ex Vivo TLR-7 Activation Is Associated with Downregulation of Mdr1b in Placental Explants

To examine whether in vivo downregulation of PGP in IMQ-treated rats was a direct result of TLR-7 signaling, we explored the response to TLR-7 activation ex vivo in rat placental explants. We challenged the explants with TLR-7 activators to examine the inflammatory response and transporter changes generated by specific stimulation of this pathway. In addition to the TLR-7 agonist, IMQ, we examined the effect of the TLR-7 agonist ssRNA40, which is a natural ligand of TLR-7. While ssRNA40 is labile and has limited stability in vivo, it is suitable for in vitro studies. We also challenged the explants with maternal serum from 6 h IMQ-treated rats, which contained inflammatory mediators produced after IMQ-mediated TLR-7 stimulation but with minimal direct effects of IMQ, as the serum levels of IMQ at 6 h were very low due to its rapid metabolism and short (~2 h) systemic half-life [42,43].

**Figure 6:**
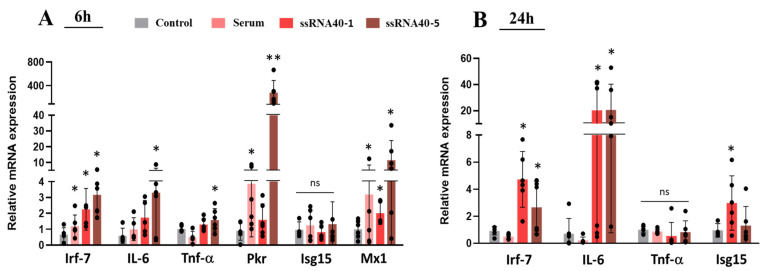
Effect of TLR-7 stimulation on transcript levels of inflammatory biomarkers in cultured rat placental explants. Explants were treated for (**A**) 6 h or (**B**) 24 h in fresh media containing serum (10%) from 6 h IMQ-treated dams, ssRNA40 (1 or 5 ug/mL), or media alone (control) and analyzed via qRT-PCR as described in methods. Levels were normalized to *Gapdh* and shown as mean ± SEM relative to control. Statistics were performed using Student’s paired *t*-test of control vs treated, as well as one-way ANOVA with Dunnet post-test. (* *p* < 0.05, ** *p* < 0.01, ns—non-significant).

We found that ssRNA40 (1 and 5 ug/mL) and serum treatment (at 10% dilution) stimulated the TLR-7 signaling pathway, as verified by induction of *Irf7* gene. Significantly increased transcript levels of proinflammatory cytokines (*Il-6*, *Tnf-a*) and IFN-α target genes were seen in the ssRNA and serum-treated explants after 6 and 24 h (Figure 6), which were similar results to those seen in vivo.

**Figure 7:**
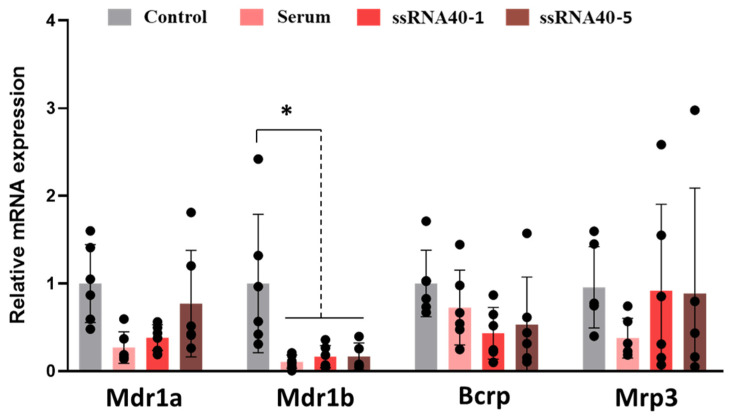
Impact of TLR-7 activation on ABC transporter expression in rat placental explants. Explants were treated in fresh media containing serum (10%) from 6 h IMQ-treated dams, ssRNA40 (1 or 5 ug/mL), or media alone (control), and transcript levels of transporters were analyzed via qRT-PCR as described in methods. Levels were normalized to *Gapdh* and shown as mean ± SEM relative to control. Statistics were performed using Student’s paired *t*-test of control vs treated, as well as one way ANOVA with Dunnet post-test. * *p* < 0.05.

Direct TLR-7 activation with ssRNA40 was found to significantly decrease the transcript levels of *Mdr1b* but not *Mdr1a*, *Bcrp, Mrp1*, or *Mrp3* (Figure 7), which was consistent with in vivo observations. Likewise, the treatment of explants with serum from IMQ-treated dams also decreased the *Mdr1b* levels but did not significantly affect the levels of the other transporters. On the other hand, while the treatment of explants with 5 ug/mL of IMQ resulted in a pronounced induction of *Irf7, Il-6*, and IFN-α target genes (Supplemental Figure S4), 2–3-fold increases in the mRNA expression of *Mdr1a/b, Bcrp, Mrp1*, and *Mrp3* were seen, which were contrary to the results seen in vivo (Supplemental Figure S4D).

## 4. Discussion

Heightened immunological responses to infectious diseases or chronic diseases during pregnancy negatively impact maternal and fetal outcomes worldwide [44]. Physiological, immunological, and hormonal changes, along with increased nutrient demands, make pregnant women and their developing fetuses more susceptible to environmental pathogens and underlying immunity disorders [25,45]. Numerous preclinical and clinical studies have determined that the TLR-7 pathway plays a pivotal role in the inflammatory processes and pathogeneses of common ssRNA viral infections such as COVID-19, HIV, and influenza, as well as autoimmune diseases such as Sjögren’s syndrome and lupus [17,21,22,46], which have high prevalence rates in women of child-bearing age. Maternal and fetal outcomes derived from those diseases include vertical transmission, intrauterine growth restriction (IUGR), preterm labor, neurodevelopmental alterations, and even neonatal death, while they predispose the mother to other pregnancy complications such as gestational diabetes and pre-eclampsia [25,44,47].

In the present work, we demonstrated that in vivo administration of IMQ to pregnant rats led to the production of proinflammatory cytokines such as IL-6, Tnf-α, and type-I interferons, all of which are consistent with TLR-7 activation. This resulted in hepatic and placental inflammation. The immune alterations observed in IMQ-treated dams are consistent with the cytokine profiles found in the sera of patients with chronic viral infections or autoimmune diseases such as COVID-19, HIV, Sjögren’s syndrome, and lupus [20,23,48,49,50]. Proteomic profiling of placentas revealed numerous IMQ-mediated alterations that are consistent with TLR-7-linked diseases [51,52,53,54]. We found altered placental levels of numerous proteins, including protease inhibitors, transcription factors, and acute phase proteins that have been reported as dysregulated in the serum proteome profiles of lupus [55] and COVID-19 patients [56,57], as well as in the placental proteome profiles of lupus patients [41] (Table S2).

Type-I interferons, particularly IFN-α, play a crucial role in the pathogeneses of both chronic viral infections and autoimmune diseases [22,49,58,59]. In the current study, a strong induction of IFN-α target genes, including *Mx1* and *Isg15*, was demonstrated in the placentas of IMQ-treated dams. A significant upregulation of their protein products was also identified in our proteomic characterization. In line with this, elevated protein levels of ISG15 and MX1 have been found in the sera of COVID-19 patients [56], while increased protein levels of MX1 were found in the placental proteome profiles of women with lupus [41]. This is consistent with other studies that have shown the implication of ISG15 and MX1 in the progression of lupus and some of its crucial pathological events, such as lymphocytopenia and lupus nephritis [59,60]. Likewise, both factors seem to be involved in inflammatory complications of COVID-19 and other chronic viral diseases [58,61,62,63]. Overall, these results demonstrate the potential of our model for examining viral and autoimmune-induced inflammation during pregnancy.

The role of TLR-7 in the regulation of drug transporters is not well understood. Here, we demonstrate that in vivo TLR-7 activation with IMQ during pregnancy leads to a decrease in the placental, hepatic, and renal expression of PGP. We found the downregulation of *Mdr1b* levels in the placentas along with the downregulation of *Mdr1a* levels in the livers and kidneys in the IMQ-treated dams. On the other hand, *Mdr1b* was induced in the liver samples. This is consistent with the fact that *Mdr1a* and *Mrd1b* demonstrate distinct patterns of tissue expression and regulation [64,65]. Others have also observed downregulation of PGP, which were associated with reduced *Mdr1a* levels, while *Mdr1b* levels either increased or remained unchanged [15,16,66,67]. Distinct promoter binding regions are found within the *Mdr1a and Mdr1b* genes, which can be uniquely regulated by endogenous and exogenous factors [65,68]. Despite this, the observed changes in the *Mdr1* transcript levels resulted in an overall significant reduction in the protein expression of PGP in the placentas, livers, and kidney.

Other inflammatory triggers have been reported to impact the tissue expression of PGP. Indeed, administration of the viral mimetic poly(I:C), a TLR-3 agonist, to pregnant rats was observed to reduce the placental expression of PGP at both the gene and protein levels, leading to increased fetal accumulation of lopinavir [15]. Similarly, treatment of HIV-1 transgenic rats with low doses of the TLR-4 agonist endotoxin was found to reduce *Mdr1a* expression and PGP protein levels in both the placenta and liver [69]. Endotoxin-mediated neuroinflammation also produces a downregulation of *Mdr1a* and PGP functionality in the brains and livers of male rats [64]. On the other hand, while adjuvant-induced arthritis was associated with both decreased hepatic expression of PGP and decreased PGP-dependent biliary excretion of rhodamine 123, an increased renal expression of PGP was seen [70]. Overall, this evidence demonstrates PGP dysregulation in response to inflammation, and our results confirm this in the context of TLR-7 activation during pregnancy.

Although TLRs activate distinct signaling pathways, they each play a crucial role in initiating immune responses. The stimulation of TLR-3, TLR-4, or TLR-7 leads to NF-κB activation, which drives the production of proinflammatory cytokines, including IL-6, TNF-α, and IL-1β [71,72]. TLR-3 specifically induces IRF3, resulting in IFN-β production [71,73,74], while TLR-7 activates IRF7, triggering a signature induction of IFN-α genes [20,36]. In our in vivo model of TLR-7 activation, we confirmed stimulation of the Irf7 and IFN-α pathways in the placenta and liver, along with elevated levels of IL-6. While IL-6 contributes to the downregulation of PGP in rat livers, it does not appear to affect PGP expression in cultured trophoblasts [75,76]. The role of IFNα in PGP regulation across various tissues, including the placenta, remains largely unexplored. A few in vitro studies investigated its effects on PGP expression and function in rat hepatocytes and Chinese hamster ovary cells, but their findings were inconclusive [77,78]. Further mechanistic studies are needed to determine whether IFN-α signaling plays a dominant role in PGP dysregulation and to clarify the molecular factors involved

To confirm the involvement of TLR-7 in PGP downregulation, we examined inflammatory biomarkers and transporters in rat placental explants following exposure to various TLR-7 activators. Exposure of the explants to IMQ resulted in elevated expression levels of *Irf7, IL-6*, and the IFN-α target gene *Mx1*, demonstrating the stimulation of the TLR-7 pathway and associated inflammation. However, IMQ treatment also increased the expression levels of *Mdr1a/b*, *Bcrp*, and *Mrp3*. As IMQ is rapidly metabolized in vivo but is neither metabolized nor eliminated in placental explants, the concentration and total exposure time to IMQ would be far greater in the explants compared to the in vivo setting and could contribute to discrepancies between ex vivo and in vivo findings. Moreover, several reports have described the induction of drug metabolizing enzymes and transporters in response to quinoline-based compounds [79,80,81]. Hence, to overcome this issue, we challenged explants with serum from IMQ-treated dams (6 h post-TLR-7 activation), and with ssRNA40, a primary ligand of TLR-7. Maternal serum from IMQ-treated dams and ssRNA40 both stimulated *Irf7* expression and triggered the associated induction of IFN-α target genes, including *Pkr, Isg15*, and *Mx1*. These treatments also caused downregulation of *Mdr1b,* whereas *Mdr1a, Bcrp*, and *Mrp3* were not affected. Thus, ex vivo stimulation of the receptor with ssRNA or maternal serum more closely mirrored our in vivo findings, leading to both TLR-7-associated responses and *Mdr1b* downregulation. Similarly, others have seen decreased PGP expression in human term placental explants treated with the TLR-3 agonist poly (I:C) [82]. In contrast, Pfeifer et al. [38] reported that although a 12 h exposure of primary cultured human cytotrophoblasts to either IMQ or ssRNA elevated the mRNA levels of proinflammatory cytokines, it did not significant impact PGP transcript levels. These discrepancies likely stem from variations in species, cell type, or culturing conditions, highlighting the need for further studies to fully characterize the impact of TLR-7 activation on the placental transporter expression.

## 5. Conclusions

This study establishes a connection between TLR-7 activation during pregnancy and PGP dysregulation. Since decreased PGP expression is often linked to reduced transport activity, this raises important questions regarding its effects on maternal drug disposition and fetal exposure to PGP substrates. Many clinically relevant drugs rely on PGP for transport, including antiviral protease inhibitors, which are approved for HIV or COVID-19 treatment during pregnancy [83,84]. Similarly, several disease-modifying antirheumatic drugs, used for lupus and other autoimmune disorders, are PGP substrates [85].

Further research is needed to determine how observed changes in PGP expression influence drug disposition and fetal exposure to clinically important PGP substrates. Additionally, studies are needed to investigate the mechanisms underlying the TLR-7 regulation of PGP expression and whether therapeutic disruption or the inhibition of TLR7 can mitigate these effects. Given the complexity of pregnancy, establishing preclinical models of maternal disease remains challenging; therefore, the IMQ model described in this study may serve as a valuable tool for examining the impact of maternal TLR-7 immune activation on fetal drug exposure.

## Data Availability

The original contributions presented in this study are included in the article/Supplementary Materials. Further inquiries can be directed to the corresponding authors.

## Supplementary Materials

The following supporting information can be downloaded at https://www.mdpi.com/article/10.3390/pharmaceutics17060741/s1. Table S1: Primers used for RT-qPCR analysis; Table S2: List of differentially modulated proteins identified by LC/MS/MS in rat placenta 24 and 48 h post-IMQ; Figure S1: Relative abundance (pg/mL) of proinflammatory cytokines, chemokines, and hormone/growth factors in the sera of imiquimod (IMQ)-treated rats (5 mg/kg, 6 h of exposure); Figure S2: Impact of imiquimod (IMQ) treatment on TLR-7 biomarkers in pregnant rats at 48 h; Figure S3: Impact of imiquimod (IMQ) treatment on ABC transporter expression in pregnant rats at 48 h; Figure S4: Effects of IMQ on TLR-7 activation, inflammation, and transporter expression in rat placental explants.

## Abbreviations

The following abbreviations are used in this manuscript:

ABC	ATP-binding Cassette
ADs	Autoimmune diseases
ANAs	Antinuclear antibodies
AOC1	Copper-containing amine oxidase
BCRP	Breast cancer resistance protein
CNDP2	Cytosolic non-specific dipeptidase or carnosine dipeptidase 2
DMEM	Dulbecco’s Modified Eagle Medium
EIF4E	Eukaryotic translation initiation factor 4E
FGA/FGG	Fibrinogen A
GD	Gestational day
GLUT-1	Glucose transporter-1
GPX-3	Glutathione peroxidase-3
GST-4	Glutathione s-transferase-4
HIV	Human immunodeficiency virus
HP	Haptoglobin
HPX	Hemopexin
HTRA1	High-Temperature Requirement A Serine Peptidase 1
I.P.	Intraperitoneal
IFN-α	Interferon alpha
IFN-γ	Interferon gamma
IG	Immunoglobulin
IL-6	Interleukin 6
IMQ	Imiquimod
IP-10	Interferon gamma-induced protein 10
IRF7	Interferon regulatory factor 7
ISG15	Interferon-stimulated gene 15
IUGR	Intrauterine growth restriction
LAMP-1	Lysosome-associated membrane glycoprotein 1
MCP-1	Monocyte Chemoattractant Protein-1
MCT-1	Monocarboxylate transporter-1
MDR	Multidrug resistance
MIP-1α	Macrophage Inflammatory Protein-1 alpha
MRP	Multidrug resistance protein
MX1	Myxovirus resistance protein 1
OAS1B	2′-5′ oligoadenylate synthetase 1B
PGP	P-glycoprotein
PKR	RNA-dependent protein kinase
poly(I:C)	Polyinosinic-polycytidylic acid
PON1	Paraoxonase 1
RAB-6A	Ras-related protein Rab-6A
RT-qPCR	Real-time quantitative polymerase chain reaction
SERPING1	Serpin Peptidase Inhibitor, Clade G, Member 1
ssRNA	Single-stranded RNA
TLR-7	Toll-like receptor 7
TNF-α	Tumor necrosis factor alpha
