# Supplementary material for: Linking TLR-7 Signaling to Downregulation of Placental P-Glycoprotein: Implications for Fetal Drug Exposure
Source: Pharmaceutics. 2025 Jun 5;17(6):741. doi: 10.3390/pharmaceutics17060741 (PMC12197206; doi:10.3390/pharmaceutics17060741)
Supplement: Supplementary file 1 [file pharmaceutics-17-00741-s001.zip › pharmaceutics-3635593-supplementary.pdf]

Activation of Toll-like Receptor (TLR)-7 is associated with Dysregulation of P-glycoprotein in a Pregnant Rodent Model of Inflammation.

Riera-Romo M., et al.

Supplementary materials

Table S1. Primers used for RT-qPCR analysis.

gapdh	Forward	5'-GCTCTCTGCTCCTCCCTGTTC-3'
	Reverse	5' -GAGGCTGGCACTGCACAA-3'
irf7	Forward	5'-CAAGGTGTATGAACTTAGCC-3'
	Reverse	5'-CTAGGGACATACCCTGTGT-3'
il-6	Forward	5'-GATGGATGCTTCCAAACTGGATAT-3'
	Reverse	5'-TCCAGAAGACCAGAGCAGATTTT-3'
tnf- α	Forward	5'-GGTCCCAACAAGGAGGAGAAGT-3'
	Reverse	5'-TGGGCCATGGAACTGATGA-3'
baff	Forward	5'-GACCGGAGGAAACAGAACAA-3'
	Reverse	5'-TGCAATCAGCTGCAGACAGT-3'
pkr	Forward	5'-GAGGATGAAAGAAACAGCCAGCT-3'
	Reverse	5'-CCCGCTATGAAATTAGATTCACGT-3'
oas1b	Forward	5'-ACAGTACGCCCTGGAGCTGCTC-3'
	Reverse	5'-CAGGTTGTAAGGAAGGCTGCTC-3'
isg15	Forward	5'-GCCATGACCTGGAACCTAAA-3'
	Reverse	5'-CTGCCTGATAAGGGCAACAC-3'
mx1	Forward	5'-CCTGAGGTAAGGCTGTGGAA-3'
	Reverse	5'-AATACGGCCCCACAAAACAC-3'
mdr1a	Forward	5'-GCAGGTTGGCTGGACAGATT-3'
	Reverse	5'-GGAGCGCAATTCCATGGATA-3'
mdr1b	Forward	5'-AAACATGGCACGTAACCAAAGTT-3'
	Reverse	5'-AAAATGTGGCCCTGTTTAATGATT-3'
bcrp	Forward	5'-TTGGACTCAAGCACAGCAAAT-3'
	Reverse	5'-ATGGAATACCGAGGCTGGTGA-3'
mrp1	Forward	5'-AGAAGGAATGTGTTAAGTCGAGGAA-3'
	Reverse	5'-CCTTAGGCTTGGGATCTT-3'
mrp3	Forward	5'-GGAGTCTACGCCACCCTAGGA-3'
	Reverse	5'-GTGAAGGCCGACAGCATGA-3'

Table S2. Differentially modulated proteins identified by LC/MS/MS in rat placenta 24 and 48h-post IMQ.

Percentage Change [#]	Protein IDs	Protein names	Gene names	24h	48h
219%	D3ZK97;P84245	Histone H3;Histone H3.3	H3f3c;H3f3b [a]	upregulated	-
176%	Q69DC0;Q69DC1	Cyclin-dependent kinase inhibitor 1C	Cdkn1c	upregulated	-
148%	P20760	Ig gamma-2A chain C region	Igg-2a	upregulated	-
146%	D3ZX01	40S ribosomal protein S4	Rps4y2 [a]	upregulated	-
142%	D4A3X3	Interferon-stimulated gene 15	Isg15 [c]	upregulated	-
113%	Q2KN99	Cytospin-A	Specc1l	upregulated	-
110%	Q5M878;Q7TMC3	Serum amyloid A protein	Hps5;Saa4 [c]	upregulated	-
87%	A0A0H2UHM3;P06866	Haptoglobin;Haptoglobin alpha chain;Haptoglobin beta chain	Hp [b, c]	upregulated	upregulated
86%	A0A0G2K2S2;P11167	Solute carrier family 2, facilitated glucose transporter member 1	Slc2a1	upregulated	-
85%	A0A0H2UHI5;A0A0G2KB85;A0A0G2K9B1;P09006	Serine protease inhibitor A3N	Serpina3n [a, b]	upregulated	-
79%	F1LN61;A0A0G2K9Y0;F1LPQ6;P20759	Ig gamma-1 chain C region		upregulated	-
64%	A0A0G2JVP4;F1LM30;F1LPW0			-	upregulated
61%	P61354;M0R7P0;D3ZTK5	60S ribosomal protein L27	Rpl27;LOC100912027;RGD1563835 [a]	upregulated	-
59%	Q6P734	Plasma protease C1 inhibitor	Serping1 [b, c]	upregulated	-
48%	Q925G0	RNA-binding protein 3	Rbm3	upregulated	-
47%	D3ZE00;A0A0G2K3A6;A0A0G2JUY4;A0A0G2K290			upregulated	-
44%	D4A9L2	Serine/arginine-rich splicing factor 1	Srsf1	upregulated	-
39%	G3V9N1;Q562B5	Serine/threonine-protein phosphatase PGAM5, mitochondrial	Pgam5	upregulated	-
38%	Q6AYT7	Monoacylglycerol lipase ABHD12	Abhd12	upregulated	-
33%	Q63016	Large neutral amino acids transporter small subunit 1	Slc7a5 [a]	upregulated	-
31%	A0A0H2UHP9;A0A0G2JT78;Q9WVB1;F1LVC3;A0A0G2K201;D3ZZP2	Ras-related protein Rab-6A	Rab6a/6b;Rab39a [a]	-	upregulated
28%	P85515	Alpha-centractin	Actr1a	-	upregulated
27%	P05371;G3V836;A0A0G2KB42	Clusterin;Clusterin beta chain;Clusterin alpha chain;Clusterin	Clu [b]	upregulated	upregulated
25%	D4A8T3	Coatomer subunit zeta-1	Copz1	-	upregulated
24%	A0A0G2K7B6;D4A6X1	Dysferlin	Dysf	upregulated	-

23%	D4A7U1	Zyxin	Zyx [c]	upregulated	-
20%	D3ZTR5;M0R785;A0A0G2K6U2	Zinc finger BED-type containing 5	Zbed5;Chchd2	upregulated	-
17%	P43278	Histone H1.0;Histone H1.0, N-terminally processed	H1f0	upregulated	-
14%	Q63396	Activated RNA polymerase II transcriptional coactivator p15	Sub1	-	upregulated
12%	Q499S4;P18588;Q499Q3;P18590;P18589	Interferon-induced GTP-binding protein Mx1	Mx1 [a, c]	upregulated	-
10%	A0A0G2K642;G3V9U2;P13437	3-ketoacyl-CoA thiolase, mitochondrial	Acaa2	downregulated	-
11%	F7FEM5			downregulated	-
14%	Q62878	3 beta-hydroxysteroid dehydrogenase/Delta 5-->4-isomerase type 4;3-beta-hydroxy-Delta(5)-steroid dehydrogenase;Steroid Delta-isomerase	Hsd3b6 [a]	downregulated	-
14%	Q3MIE4	Synaptic vesicle membrane protein VAT-1 homolog	Vat1	downregulated	-
15%	D4A269;P62959	Histidine triad nucleotide-binding protein 1	Hint1	-	downregulated
17%	Q6AYK6	Calcyclin-binding protein	Cacybp [c]	downregulated	-
17%	Q9JKB7;Q9WTT6	Guanine deaminase	Gda [c]	downregulated	-
17%	F1M7Q5;O09178;P10759	AMP deaminase 3	Ampd3	downregulated	-
17%	M0RCH6;D4A9Z8	Charged multivesicular body protein 4b	Chmp4b;Chmp4bl1	downregulated	-
22%	P55159	Serum paraoxonase/arylesterase 1	Pon1	downregulated	-
24%	B1WC67	Mitochondrial adenyl nucleotide antiporter	Slc25a24	downregulated	-
28%	P10960;F7EPE0	Sulfated glycoprotein 1	Psap	downregulated	-
33%	Q4FZU6	Annexin A8	Anxa8 [a]	downregulated	-
37%	A0A0G2JW98;P06757;Q5XI95	Alcohol dehydrogenase 1	Adh1	downregulated	-
37%	D3Z8D7;A0A0G2K743;P62856;D3ZJ54	40S ribosomal protein S26	LOC100361854;Rps26;RGD1565117	-	downregulated
39%	F1MAB9;A0A0G2K865	Tumor protein D52	Tpd52	downregulated	-
39%	A0A1W2Q627;P63074	Eukaryotic translation initiation factor 4E	Eif4e [a, c]	downregulated	-
40%	Q6Q0N1	Cytosolic non-specific dipeptidase	Cndp2 [c]	downregulated	-
40%	P84083	ADP-ribosylation factor 5	Arf5 [a]	downregulated	-

41%	Q5U318	Astrocytic phosphoprotein PEA-15	Pea15 [c]	downregulated	-
41%	D4A771;D3ZZN4;P04646;F1M0T2;A0A096P6M4	60S ribosomal protein L35a	LOC100362049;LOC100362338;Rpl35a	-	downregulated
41%	P14562	Lysosome-associated membrane glycoprotein 1	Lamp1 [a]	-	downregulated
43%	A0A0G2K7Y0;Q63135	Complement component receptor 1-like protein	Cr1l	downregulated	-
43%	Q62658	Peptidyl-prolyl cis-trans isomerase	Fkbp1a [c]	-	downregulated
43%	P08050	Gap junction alpha-1 protein	Gja1	downregulated	-
47%	P62718;F1M0K6	60S ribosomal protein L18a	Rpl18a;RGD1565566	downregulated	-
53%	Q9R006	Prolactin-7A2	Prl7a2	downregulated	-
57%	P51886	Lumican	Lum [a]	downregulated	-
59%	Q9QZK5	Serine protease HTRA1	Htra1 [c]	downregulated	-
60%	Q9EPT8	Chloride intracellular channel protein 5	Clic5 [a]	downregulated	-
62%	P31211	Corticosteroid-binding globulin	Serpina6	downregulated	-
63%	Q8CHN6	Sphingosine-1-phosphate lyase 1	Sgpl1	downregulated	-
65%	D4A412;P62912	60S ribosomal protein L32	LOC688684;Rpl32	-	downregulated
68%	P10959;D3ZGK7;G3V822;A0A0G2K3Z4;A0A0G2JY66	Carboxylesterase 1C;Carboxylic ester hydrolase	Ces1c	-	downregulated
68%	Q6AYS8	Estradiol 17-beta-dehydrogenase 11	Hsd17b11 [a]	downregulated	-
70%	D4A6E3;A0A0G2JUW7		LOC100911833	-	downregulated
73%	Q63545	Decidual prolactin-related protein	Prl8a2	downregulated	-
77%	P20762	Ig gamma-2C chain C region		downregulated	-
80%	D3ZKU5;D3ZX87;P62902;D3ZK34;D3ZDE9;D3ZU04;D3Z8W1;D4ACQ2;D4ABZ9;D3ZZ60;A0A0G2K7E3;D3ZVY6	60S ribosomal protein L31	RGD1564839;LOC100910017;Rpl31;RGD1565894;RGD1562055;LOC102550734;LOC680384;LOC690384;Rpl31l4;LOC681356;LOC100359986	-	downregulated
83%	B1PLB1;B1PLB2		Cd34	downregulated	-
85%	B5DEP7;A0A0G2K3V8		LOC100911683	downregulated	-
94%	G3V8G2	Proteasome 26S Subunit, Non-ATPase 5	Psmd5 [c]	downregulated	-
95%	Q63636;M0R5I8	Granzyme C	Gzmc	downregulated	-
96%	E9PU13	Sorting nexin 4 endosomal protein	Snx4	downregulated	-
97%	P13852	Major prion protein	Prnp	downregulated	-
98%	F1LUD3	AHNAK nucleoprotein-2	Ahnak2	downregulated	-
104%	A0A0H2UHR7;D3ZHA0	Filamin-C	Flnc [a]	downregulated	-

105%	P47967;G3V7N2	Galectin-5;Galectin	Lgals5	downregulated	-
107%	P23928	Alpha-crystallin B chain	Cryab	downregulated	-
115%	P05942	Protein S100-A4	S100a4 [c]	downregulated	-
119%	P06238	Alpha-2-macroglobulin	A2m [b]	downregulated	-
133%	P05943	Protein S100-A10	S100a10	downregulated	-
145%	Q6P725;P48675;G3V8Q2;G3V7S2;F1LRZ7;P23565;P19527;P16884	Desmin	Des [a]	downregulated	-
151%	P36633;Q498N2;A0A0G2JXH5	Amiloride-sensitive amine oxidase [copper-containing];Amine oxidase	Aoc1 [a, c]	downregulated	-
154%	A0A0G2JY70;A0A0H2UHG7;D3ZZK1;A0A0G2K6L6;P60868	40S ribosomal protein S20	LOC100359563;Rps20;LOC100909911;RGD1562725;RGD1563124;LOC100364116	-	downregulated
172%	P20717;F1LPA6	Protein-arginine deiminase type-2	Padi2	downregulated	-
204%	A0A140TA89;P50442	Glycine amidinotransferase, mitochondrial	Gatm	downregulated	-
259%	Q05982	Nucleoside diphosphate kinase A	Nme1 [a]	downregulated	-

[#] Only proteins with percentage change ≥ 10% are shown; [a] Proteins reported as differentially modulated in placenta of lupus patients [1]; [b] Proteins reported as differentially modulated in serum of lupus patients [2]; [c] Proteins reported as differentially modulated in serum of COVID-19 patients [3].

1. Pollinzi, A., et al., *Decreased expression of P-glycoprotein in the placenta of women with autoimmune disease.* 2025. **53**(2): p. 100031.

2. Madda, R., et al., *Plasma proteomic analysis of systemic lupus erythematosus patients using liquid chromatography/tandem mass spectrometry with label-free quantification.* PeerJ, 2018. **6**: p. e4730.

3. Babačić, H., et al., *Comprehensive proteomics and meta-analysis of COVID-19 host response.* 2023. **14**(1): p. 5921.

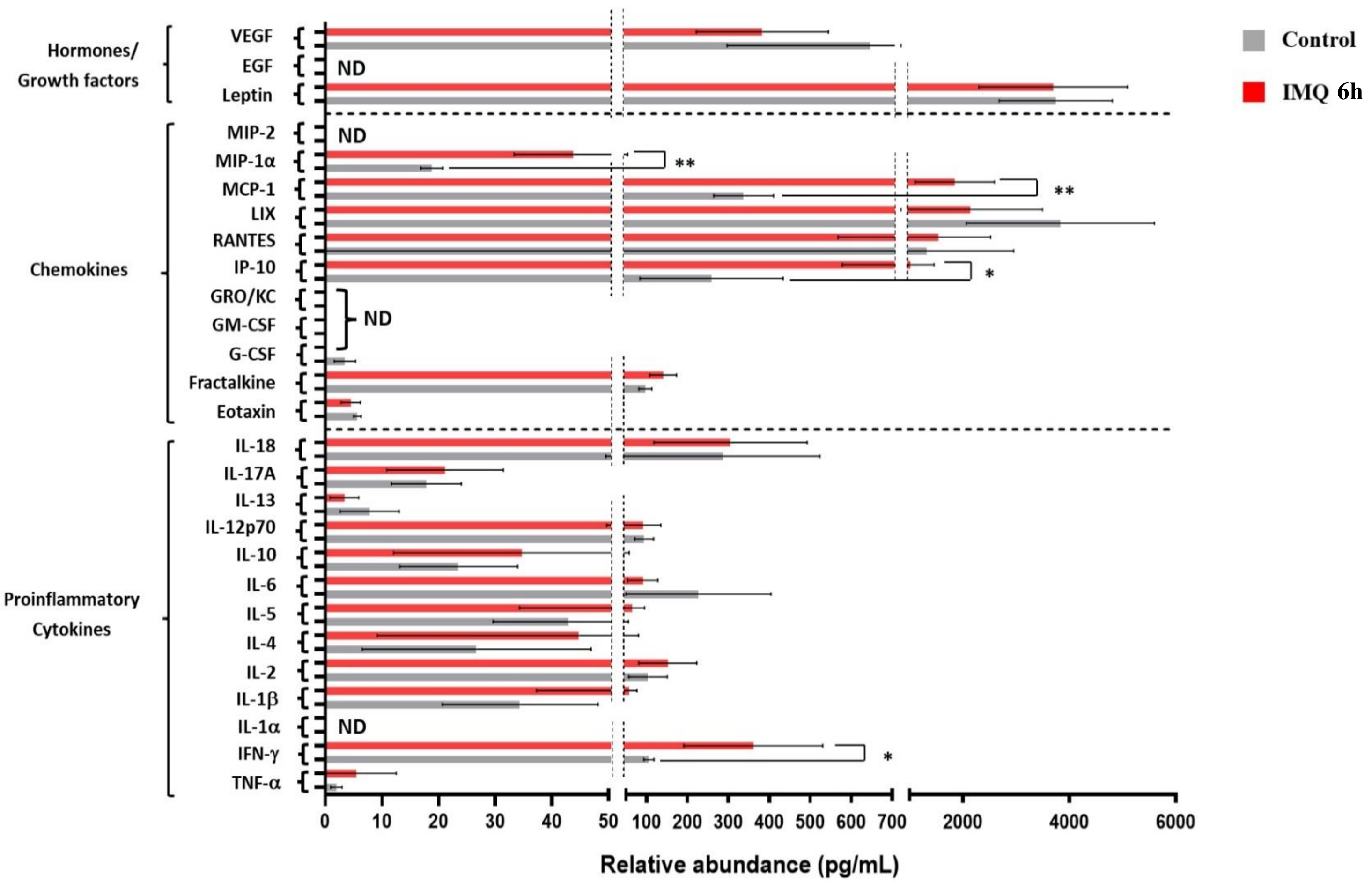

Figure S1. Relative abundance (pg/mL) of proinflammatory cytokines, chemokines and hormone/growth factors in the serum of imiquimod (IMQ) treated rats (5mg/kg, 6h-post injection). The protein levels were measured by the Rat Cytokine 27-Plex Discovery Assay® from Eve Technologies (Calgary, AB Canada). The values are expressed as the average ± SEM relative to saline controls. ND refers to certain proteins with non-detectable levels in the samples. Statistical significance was determined using Student's unpaired t test (*P < 0.05, **P < 0.01).

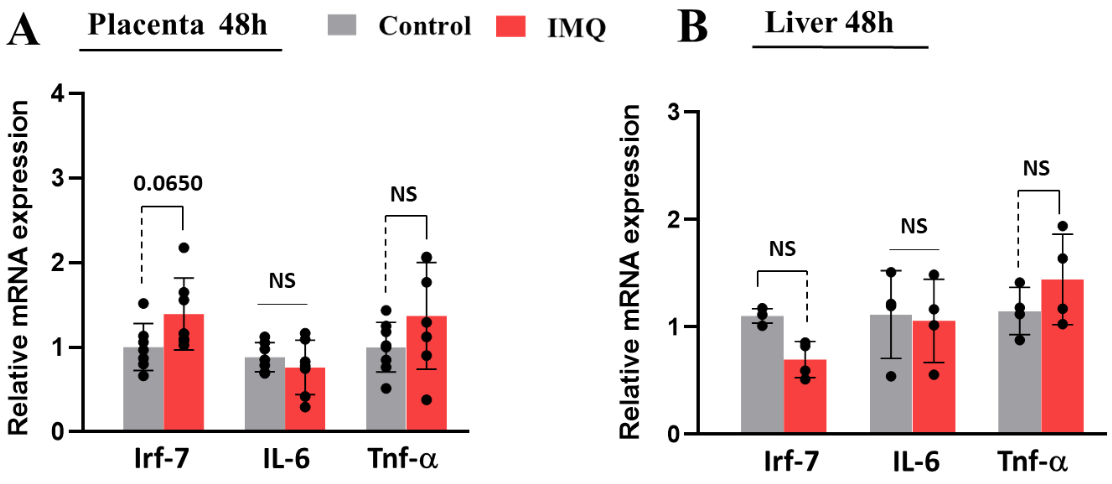

Figure S2. Impact of imiquimod (IMQ) treatment on TLR-7 biomarkers in pregnant rats at 48h. Transcript levels of Irf7, IL-6 and Tnf-a in placenta (A) and liver (B) 48h post IMQ. Animals were injected on GD 14 with IMQ (5 mg/kg) or saline (control). Expression of proinflammatory biomarkers was measured via qRT-PCR as described in methods. Statistical significance was determined using Student's unpaired t test (* $P < 0.05$).

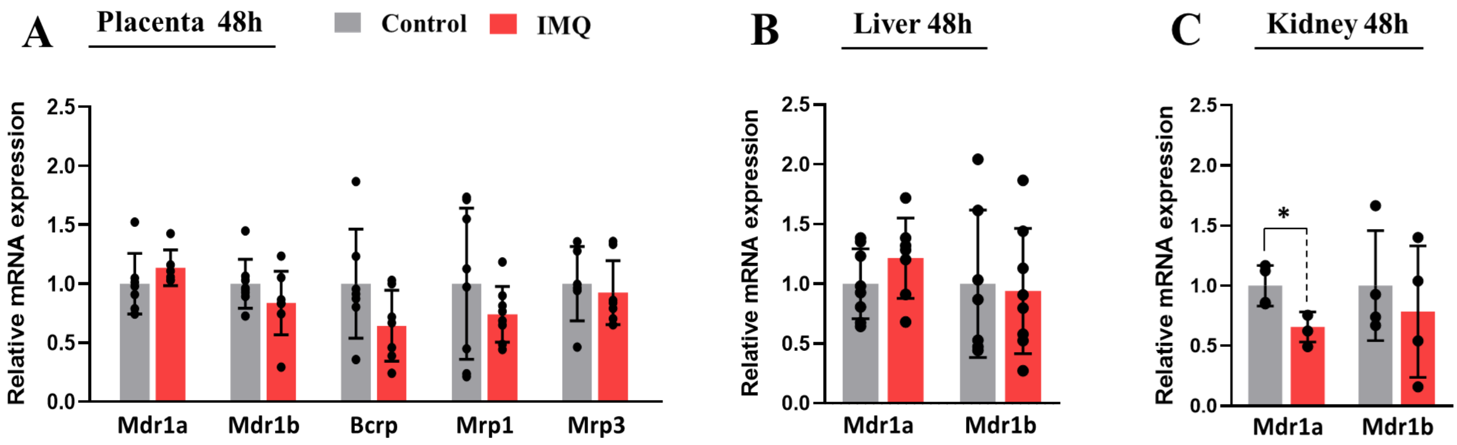

Figure S3. Impact of imiquimod (IMQ) treatment on ABC transporter expression in pregnant rats at 48h. Transcript levels of Bcrp, Mdr1a and Mdr1b [P-gp], Mrp1 and Mrp3 in placenta 48h post IMQ (A). Transcript levels of Mdr1a and Mdr1b in liver (B) and kidney C), 48h after IMQ injection. Animals were injected on GD 14 with IMQ (5 mg/kg) or saline (control). Expression of transporters was measured via qRT-PCR as described in methods. Statistical significance was determined using Student's unpaired t test (* $P < 0.05$).

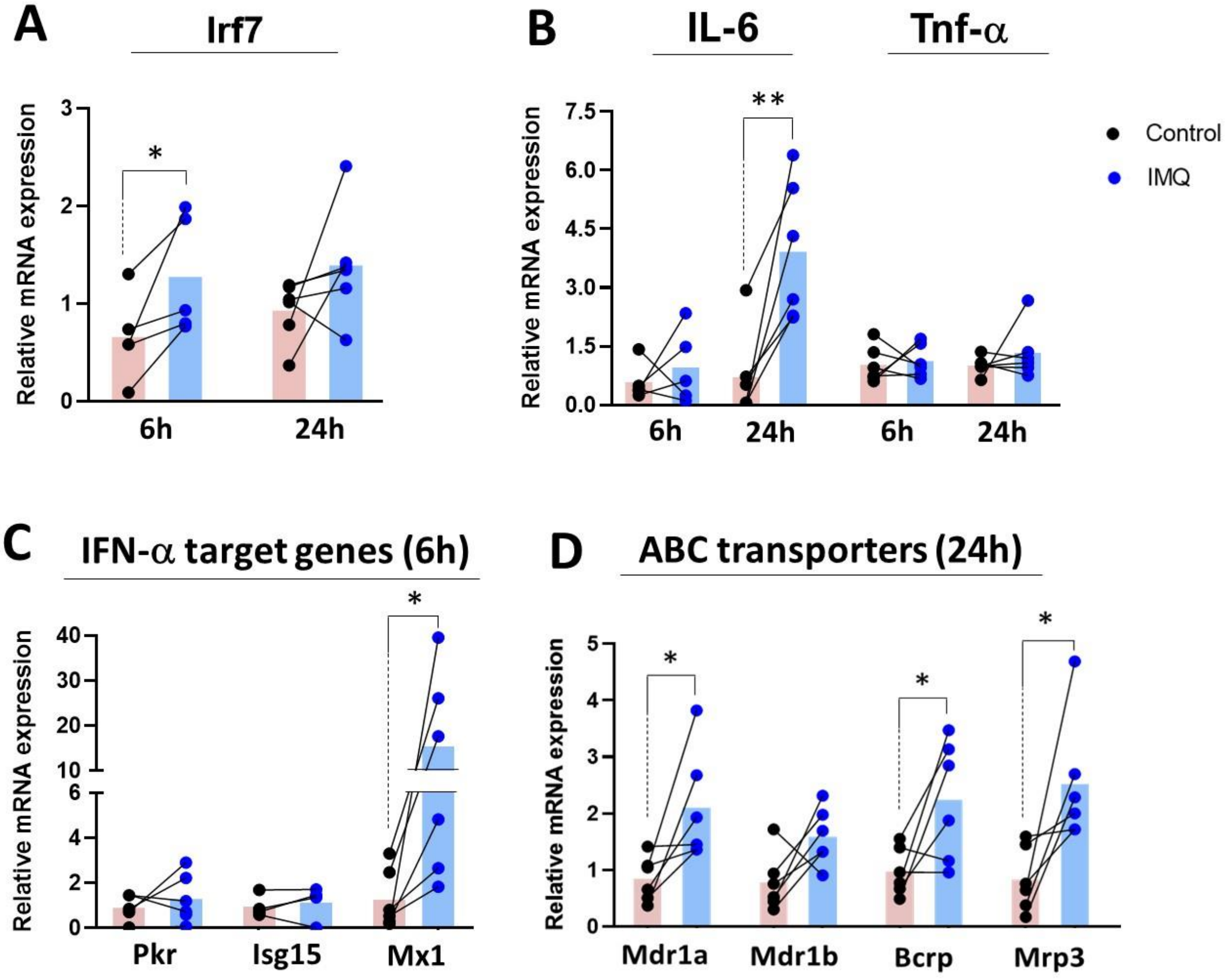

Figure S4. Effects of IMQ on TLR-7 activation, inflammation and transporter expression in rat placental explants. Transcript levels of *Irf7* **(A)**, *IL-6* and *Tnf-a* **(B)**, the interferon alpha target genes, *Pkr, Isg15* and *Mx1* **(C)** and the main drug transporters *Mdr1a/b* (P-gp), Bcrp and Mrp3 **(D)**. Explants were stablished using placentas from healthy GD 19 pregnant rats and treated with IMQ (5 ug/mL) dissolved in culture media. Untreated explants just incubated with fresh culture media were the controls. Explants were treated for 6 and 24h, total RNA was extracted, and the expression was measured using RT-qPCR, normalized to *Gapdh*, and expressed as the average ± SEM relative to controls. Statistical significance was determined using Student's paired t test (* $P < 0.05$, ** $P < 0.01$).
